# Vascular plant species diversity of Mt. Etna (Sicily): endemicity, insularity and spatial patterns along the altitudinal gradient of the highest active volcano in Europe

**DOI:** 10.7717/peerj.9875

**Published:** 2020-11-18

**Authors:** Saverio Sciandrello, Pietro Minissale, Gianpietro Giusso del Galdo

**Affiliations:** Department of Biological, Geological and Environmental Sciences, University of Catania, Catania, Italy

**Keywords:** Diversity, Elevation gradient, Endemic flora, Hot spot analysis, Isolation, Mediterranean endemic flora, Plant life forms, Spatial patterns

## Abstract

**Background:**

Altitudinal variation in vascular plant richness and endemism is crucial for the conservation of biodiversity. Territories featured by a high species richness may have a low number of endemic species, but not necessarily in a coherent pattern. The main aim of our research is to perform an in-depth survey on the distribution patterns of vascular plant species richness and endemism along the elevation gradient of Mt. Etna, the highest active volcano in Europe.

**Methods:**

We used all the available data (literature, herbarium and seed collections), plus hundreds of original (G Giusso, P Minissale, S Sciandrello, pers. obs., 2010–2020) on the occurrence of the Etna plant species. Mt. Etna (highest peak at 3,328 mt a.s.l.) was divided into 33 belts 100 m wide and the species richness of each altitudinal range was calculated as the total number of species per interval. In order to identify areas with high plant conservation priority, 29 narrow endemic species (EE) were investigated through hot spot analysis using the “Optimized Hot Spot Analysis” tool available in the ESRI ArcGIS software package.

**Results:**

Overall against a floristic richness of about 1,055 taxa, 92 taxa are endemic, of which 29 taxa are exclusive (EE) of Mt. Etna, 27 endemic of Sicily (ES) and 35 taxa endemic of Italy (EI). Plant species richness slowly grows up to 1,000 m, then decreases with increasing altitude, while endemic richness shows an increasing percentage incidence along the altitudinal gradient (attributed to the increased isolation of higher elevation). The highest endemic richness is recorded from 2,000 up to 2,800 m a.s.l., while the highest narrow endemic richness (EE) ranges from 2,500 up to 2,800 m a.s.l. Life-form patterns clearly change along altitudinal gradient. In regard to the life-form of the endemics, the most represented are the hemicryptophytes, annual plants (therophytes) are prevailing at lower altitudes and show a decreasing trend with increasing elevation, while chamaephytes are featured by an increasing trend up to 3,100 m of altitude. Furthermore, the results of the hotspot analysis emphasize the high plant conservation priority areas localized in oro-mediterranean (1,800–2,400 m s.l.m.) and cryo-mediterranean (2,400–2,800 m) bioclimatic belts, in correspondence of the oldest substrates of the volcano.

**Conclusions:**

High plant speciation rate caused by increasing isolation with elevation is the most plausible explanation for the largest active volcano in Europe. The high degree of endemic species on Mt. Etna is linked to its geographical, geological and climatic isolation, all important drivers of speciation acting on the population gene flows. The hot spot map obtained represents a useful support for help environmental decision makers to identify priority areas for plant conservation.

## Introduction

Elevational gradient strongly affects species diversity and spatial patterns (*Allen & Gillooly, 2006; Buira, Aedo & Medina, 2017*). According to literature, two main patterns of species richness elevation gradient are recognized. The first one concerns the plant species number (total vascular flora) which decreases with elevation, the second one is a *hump-shaped* pattern, with a diversity peak at mid-elevations (Rahbek, 2005). But concerning the endemic component is possible an *increase* of endemism with elevation attributed to growing isolation of higher elevation (Kessler, 2002) or a *unimodal* curve in relation with peak of richness somewhere in the middle of the gradient (*Vetaas & Grytnes, 2002; Kluge & Kessler, 2006; Zhang et al., 2009*). Several researches about this topic were mainly focused on the large mountains of the world (*Vetaas & Grytnes, 2002; Kessler, 2000; Kessler, 2002; Zhang et al., 2009; Wang, Tang & Fang, 2007; Baniya et al., 2012; Acharya, Vetaas & Birks, 2011*) and on tropical islands (*Bachman et al., 2004; Cano-Ortiz et al., 2016*), while the Mediterranean islands few or no attention received, with the exception of two recent papers on Crete and Pelopennese (*Trigas et al., 2012; Trigas, Panitsa & Tsiftasis, 2013*).

Also plant life forms, as a morpho-ecological adaptation to certain environmental conditions (Odland, 2009), may be used to indicate particular climate features, biogeographic regions, major biomes of the world and other environmental differences especially in regions with a seasonal climate (Raunkit4ht=æAÁr, 1934; Klimeš, 2003). Life-form patterns along altitudinal gradient have been used for a better interpretation of vegetation and species richness in relation to environmental gradients (*Mahdavi, Akhani & Van der Maarel, 2013*).

Sicily is considered as one of the main biodiversity hot-spots in the Mediterranean Region (*Médail & Quézel, 1997; Médail & Quézel, 1999; Myers et al., 2000; Médail & Diadema, 2009*) with about 3,250 native and naturalized taxa (species and subspecies), 325 of them narrow endemic of Sicily (*Raimondo, Domina & Spadaro, 2010*). Within the island, it is possible to highlight an endemic component diversified mainly for geological districts underlining its biogeographic complexity (Brullo et al., 2011; Sciandrello et al., 2015). Mt. Etna, located in eastern Sicily, as a geologically recent volcano (Late Quaternary), is very interesting for studying plant colonization processes and speciation mechanisms which are favoured by its important altitudinal development (highest peak at 3,328 m a.s.l.), geographic isolation (i.e insular mountain system causing a “double insularity”), geo-lithological isolation, and, last but not least, the incessant volcanic activity leading to a continuous creation of new, bare land. The occurrence of a rich pool of narrow (neo-)endemic taxa, mostly occurring at high altitudes, can be seen as one of the most striking consequence of such features.

The main aim of this study is to perform an in-depth survey on the distribution patterns of vascular plant species richness and endemism along the elevational gradient of Mt. Etna, the highest active volcano in Europe and one of the highest Mediterranean mountains in order to define a plant zonation model for the Mediterranean area. This topic is particularly relevant in Mediterranean area where isolated systems (islands, archipelagos, and high mountains) favor the onset of endemism related not only to geographic isolation but also to the altitudinal gradient (Odland & Birks, 1999; Kallimanis et al., 2010; Trigas, Panitsa & Tsiftasis, 2013; Molina-Venegas et al., 2015; Buira, Aedo & Medina, 2017; Noroozi et al., 2019). However, protection and conservation strategies need more specific information to identify areas with high conservation priority or more vulnerable to the ongoing climatic change. For this purpose, 29 narrow endemic species of the Etna Volcano (EE) were investigated through hot spot analysis using GIS (Geographic Information Systems). Our main purpose is to show and clarify the spatial patterns of plant species richness along the full altitudinal gradient of Etna Mount, to identify areas with a high density of endemism, and finally to compare Etna plant diversity with that of other Mediterranean territories in order to understand if volcanic activity affects floristic diversity. Moreover, this study aims at: (1) producing a complete and updated inventory of the vascular flora; (2) analysing the distribution of life forms along the altitudinal gradient; (3) identify hotspots of narrow endemic species.

## Methods

### Study area

Mount Etna is a huge polygenic basaltic volcano covering a broad sub-rounded surface of 1,178 Km^2^ from the sea level, along the Ionian coast of Sicily, up to 3,328 m a.s.l., thus being the highest peak of Sicily and the highest active volcano of Europe. It is characterized by an almost continuous eruptive activity from its summit craters and fairly frequent lava flows from lateral fissures. Its origin is quite recent (late Quaternary) with several changes in its edification. In particular, after an earlier phase of scattered and discontinuous volcanic activity occurring about 500 ka and 330 ka ago, the volcanism in Mt. Etna region was concentrated along the Ionian Sea coast between 220 and 121 ka ago (Branca et al., 2011; Branca, Coltelli & Groppelli, 2011; De Beni et al., 2011). According to *Branca & Ferrara (2013)*, about 80% of the volcanic products were erupted only in the past 110 ka due to the stabilization of the magma source and from 15,000 years ago, the younger volcanics mantled most of the previous edifice (88% of the area) with a widespread cover of lava flow fields and pyroclastc deposits (*Branca et al., 2011a; Barreca, Branca & Monaco, 2018*). This, joined to a general process of tectonic uplifting, sometimes broken by the subsidence related to flank sliding of this volcanic edifice (*Branca et al., 2014*), contributes to understand the present structure of Etna volcano but also to highlights relevant constraints and evolutionary chances for the plants colonizing this mountain in which soils are rejuvenated quite frequently, especially at higher elevations, not only with lava flows but above all with falls of volcanic ash and tephra.

Regarding the climate, according to the bioclimatic classification proposed by *Rivas-Martinez (1993)* and Rivas-Martinez, Penas & Diaz (2004), Mt. Etna is characterized by a Mediterranean pluviseasonal oceanic bioclimate very diversified in relation to the altitude and exposure, considering also that the eastern slope is facing the Ionian Sea and then benefits from the humid air currents, and more precipitation than the western slope. Thermotypes range from the low thermomediterranean to the lower cryomediterranean while ombrotypes range from the semiarid to the upper hyperhumid (*Bazan et al., 2015*). It is relevant to underline that in Sicily the lower cryomediterranean and upper oromediterranean belts exclusively occur on Mt. Etna.

### Floristic data sources

This research is based on two fundamental elements: a complete checklist of the Etnean vascular flora and the list of endemic species as complete and updated as possible. Furthermore, it was necessary to provide accurate distribution data in order to be able to establish the altitudinal range for each taxon. Currently, an updated flora of Mt. Etna doesn’t exist and therefore it was necessary to draw up a preliminary control list. Our survey is founded, according to Sciandrello et al. (2015), on literature and herbarium data (CAT, PAL, NAP, FI; acronyms according to Thiers, 2013) increased by many field investigations carried out in the last twenty years all around the volcano. In particular, the huge collection of F. Tornabene, founder of the Botanical Garden of the University of Catania, was verified. It consists of ca. 11,000 herbarium specimens. Several distribution data of the modern collections (the so called “Erbario Generale Moderno”) were also obtained. All reports regarding Mt. Etna were considered (*Tornabene, 1889–1892; Strobl, 1880*), as well as the main floristic surveys about the whole island (*Gussone, 1843; Gussone, 1844; Gussone, 1845; Lojacono Pojero, 1888–1909; Giardina, Raimondo & Spadaro, 2007*) or Italy (*Fiori, 1925–1929; Pignatti, 1982; Pignatti, 2017–2019; Conti et al., 2005; Peruzzi, Conti & Bartolucci, 2014; Bartolucci et al., 2018*) and a number of recently published floristic and taxonomical contributions (*Brullo & Siracusa, 1994; Brullo et al., 2013a; Brullo, Pavone & Salmeri, 2013; Brullo et al., 2015; Gottschlich, Raimondo & DiGristina, 2013; Gianguzzi et al., 2014; Gianguzzi, Cusimano & Romano, 2014b*). Fundamental was the collection of floristic observations, sampled during the last twenty years all around the volcano in the form of phytosociological relevés, georeferenced floristic records, field notes, etc. Moreover, several papers, regarding the plant communities of this territory, were consulted (*Brullo et al., 1999; Brullo, Giusso del Galdo & Guarino, 2001; Brullo, Marcenò & Siracusa, 2004; Brullo et al., 2005; Brullo et al., 2007; Brullo et al., 2008; Brullo et al., 2010; Brullo et al. 2012; Brullo et al., 2013b; Brullo & Marcenò, 1985a; Brullo & Marcenò, 1985b; Brullo & Siracusa, 2000; Di Benedetto, 1981; Furnari & Scelsi, 1993; Gianguzzi, Cusimano & Romano, 2014b; Giusso del Galdo & Brullo, 2012; Grillo, 1975; Maugeri, 1980; Maugeri et al., 1979; Poli, 1965; Poli & Grillo, 1975; Poli & Maugeri, 1974; Poli, Lo Giudice & Ferlito, 1979*). More than 2,000 phytosociological published relevés were examined. As concerns the herbarium material, but also for the phytosociological relevés without any explicit altitudinal indication, but just provided with the locality (i.e toponym), elevation was determined by consulting topographic maps at 1:25,000 or 1:50,000 provided by the I.G.M. (Istituto Geografico Militare–Military Geographic Institute). Combining this huge amount of data, also checking the taxonomic and nomenclatural correspondences, it was possible to give a reliable distribution and altitudinal range for each species. Taxonomic nomenclature follows Giardina, Raimondo & Spadaro (2007) and Pignatti (2017-2018).

Mt. Etna (0–3,328 m), according to Trigas, Panitsa & Tsiftasis (2013), was divided into 33 belts 100 m wide and the plant diversity of each altitudinal belt was calculated as the total number of species per interval. All species were considered having a continuous distribution between their minimum and maximum elevation limits. Area of each altitudinal belt was calculated using digital elevation models in ArcGIS 10.3 (3D Analyst).

In order to identify areas with high plant conservation priority, 29 narrow endemic species (mapped by 93 verified points of occurence) of the Etna Volcano (EE) were investigated through hot spot analysis using the “Optimized Hot Spot Analysis” tool available in the ESRI ArcGIS^®^ software package. This tool, using the Getis-Ord Gi* statistic, identifies statistically significant spatial clusters of high values (hot spots) and low values (cold spots) of endemism presence. According to *Getis & Ord (1992)*, the Gi * statistic is used to measure the degree of association from a concentration of weighted points.

In order to do a useful chorological classification of the endemic taxa, the following groups and relative acronyms are recognized: (1) endemic to Etna (EE), with a narrow distribution area exclusive of Mt. Etna, (2) endemic to Sicily (ES) with a distribution area restricted to Sicily, and (3) endemic to Italy (EI), with a distribution area restricted to the whole Italian territory. Plant life forms were defined according to Raunkit4ht=æAÁr’s classification (1934), based on the position of renewing buds in relation to the ground soil. Simple scatter plots were used to show the patterns of species richness and endemism along the elevational gradient of Mt. Etna. Simple regression analyses were used to correlate: life form, total vascular flora, total endemism, and narrow endemic species to log-area of each elevational interval. Regression analyses, generalized linear models, were performed using the statistical package Past Version 2.17. Furthermore, in order to compare the overall flora of Etna with that of neighboring territories, some mountain and island areas of the Mediterranean have been selected, taking into account the availability of floristic data, surfaces not too different from the study area and substrate types. Spearman rank correlation coefficients were used to clarify the relationships between plant species richness and endemic species, as well as between species richness/endemic species and area. A *p*-value of 0.05 was taken as indicating a statistically significant correlation.

## Results

### Species richness and endemism

**Figure 1:**
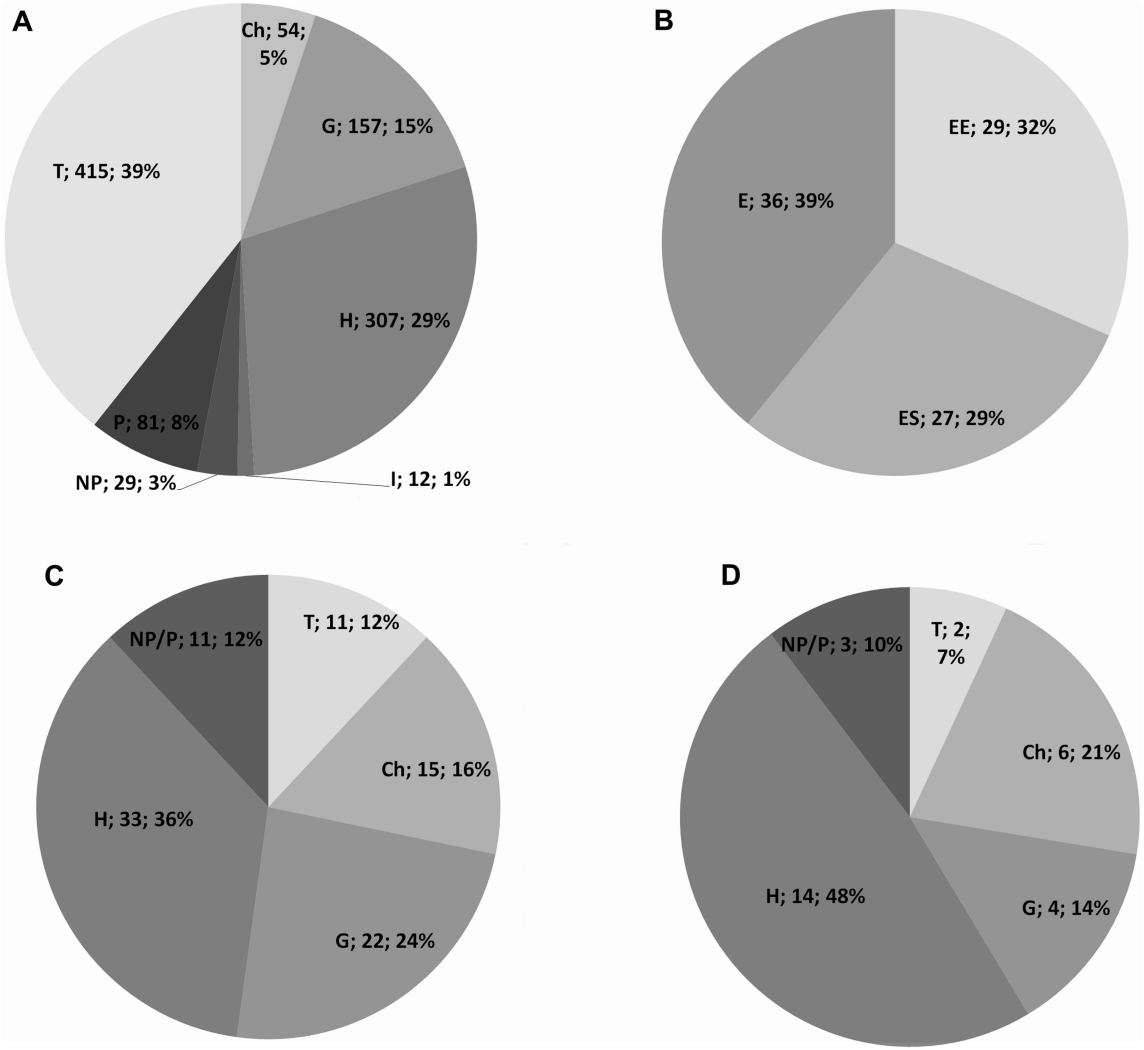
Life form of the Etna vascular flora. (A) Life form spectrum of the entire vascular flora; (B) Etnean endemic vascular flora; (C) life form spectrum of the total endemic species; (D) life form in the narrow endemic.

Based on the collected data, the vascular flora of Mt. Etna counts for 1055 taxa, belonging to 121 families (Table S1). The most represented are *Asteraceae* (143 taxa, 14%), *Poaceae* (111 taxa, 11%), *Fabaceae* (83 taxa, 8%), *Caryophyllaceae* (54 taxa, 5%), and *Orchidaceae* (52 taxa, 5%). The life form spectrum of the vascular flora indicates the prevalence of therophytes (39%), followed by hemicryptophytes (29%) and geophytes (15%) (Fig. 1A). The Etnean endemic vascular flora accounts for 92 taxa (ca. 8,7%), that is 29 narrow endemic (EE) of Mt. Etna, 27 endemic of Sicily (ES) and 36 taxa endemic of Italy (EI) (Fig. 1B). The narrow endemics are mainly localized within the oro-mediterranean and cryo-mediterranean bioclimatic belts, such as *Adenocarpus bivonii*, *Allium aetnense*, *Anthemis aetnensis*, *Astragalus siculus*, *Bellardiochloa variegata* subsp. *aetnensis*, *Betula etnensis*, *Cerastium tomentosum* var. *aetneum, Erysimum etnense*, *Galium aetnicum, Hieracium pallidum* subsp. *aetnense*, *Rubus aetnicus*, *Rumex aetnensis*, *Scleranthus aetnensis, S. vulcanicus*, *Senecio glaber*, *S. aethnensis*, *Sternbergia colchiciflora* subsp. *etnensis*, *Viola aethnensis subsp. aethnensis.*

Hemicryptophytes are the most common life form of the whole endemic flora (39%), followed by geophytes (25%), chamaephytes (15%), therophytes (12%) and nano-phanerophytes (12%) (Fig. 1C). The most represented families of the total endemic flora are *Asteraceae* (21%), *Caryophyllaceae* (9%), and *Orchidaceae* (8%). The most dominant life form in the narrow endemic (EE) are hemicryptophytes (48%) and chamaephytes (21%), followed by geophytes (14%), nano-phanerophytes (10%), and therophytes (7%) (Fig. 1D), with the most represented families *Asteraceae* (24%) and *Caryophyllaceae* (14%).

### Life form distribution patterns

**Figure 2:**
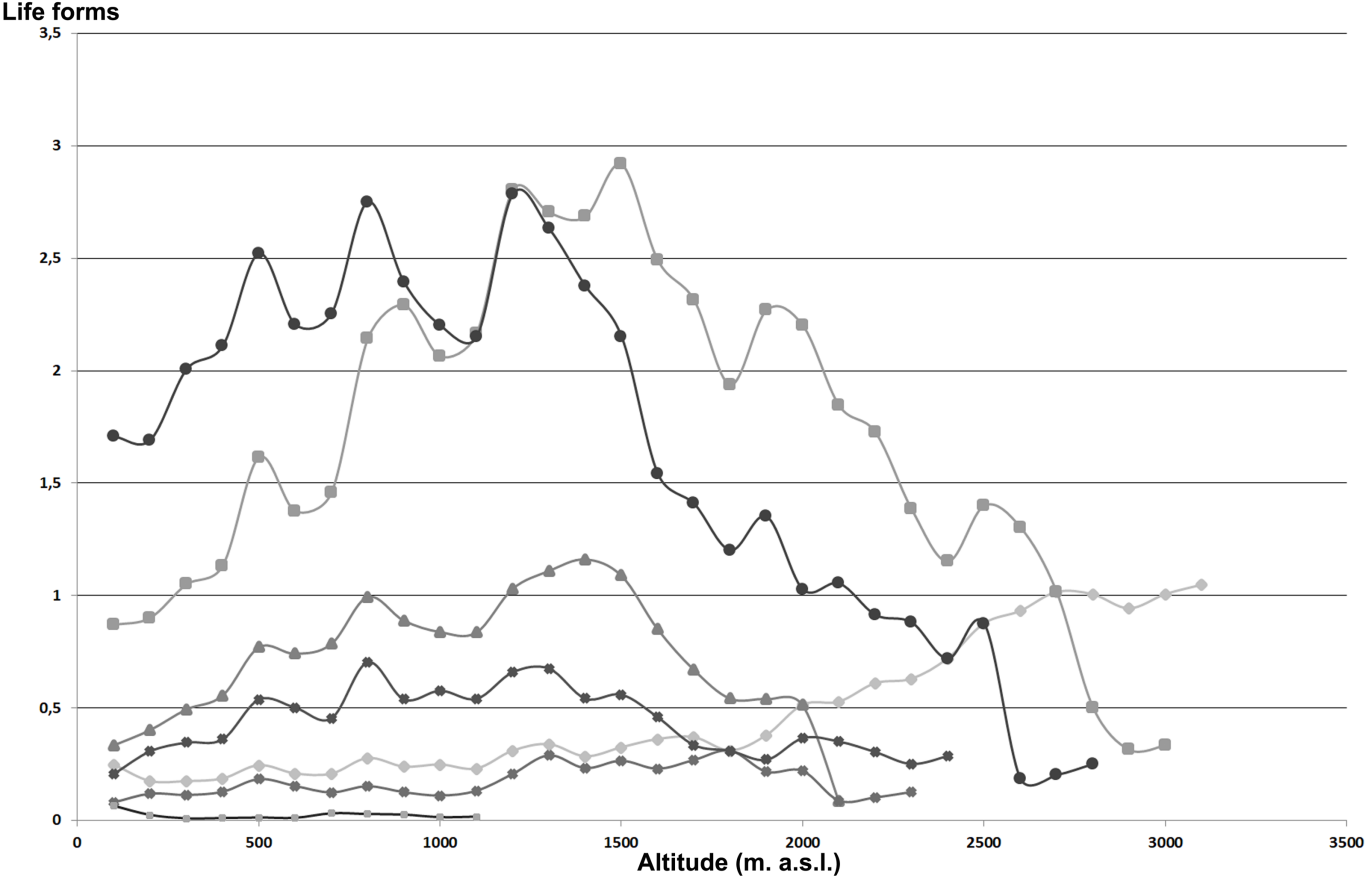
Life form of the Etna vascular flora in relation to altitude. Area/richness species ratio for each altitude range expressed as a percentage. Chamaephytes (rhombus). hemicryptophytes (square). geophytes (triangle). nanophanerophytes (× symbol). phanerophytes (asterisk). terophytes (circle). hydrophytes (+ symbol).

Life form distribution patterns along the altitudinal gradient of the whole Etnean flora are shown in Fig. 2 as percentage of each life-form category per altitudinal interval. Annual plants with 39% are the dominant life form and tend to increase up to 1,200 m a.s.l., and then they decrease. Hemicryptophytes with an average of 29% behave similarly to the therophytes with a peak at 1,500 m a.s.l. Geophytes (15%) show an increasing trend up to 1,400 m a.s.l. Chamaephytes with an average of 5% show a gradual increase with altitude, but failing to reach beyond 3,100 m a.s.l. In particular, *Astragalus siculus*, the most important species, for physiognomy and number of individuals, at high altitude, have an obvious peak between 2,000 and 2,700 m a.s.l. Phanerophytes, with an average of 8%, show a peak between 800–1,200 m a.s.l., while nano-phanerophytes (3%) show a slight increase with altitude. Above 2,400 m a.s.l., no (nano-)phanerophyte is found.

### Elevational gradient of species richness/endemism

**Figure 3:**
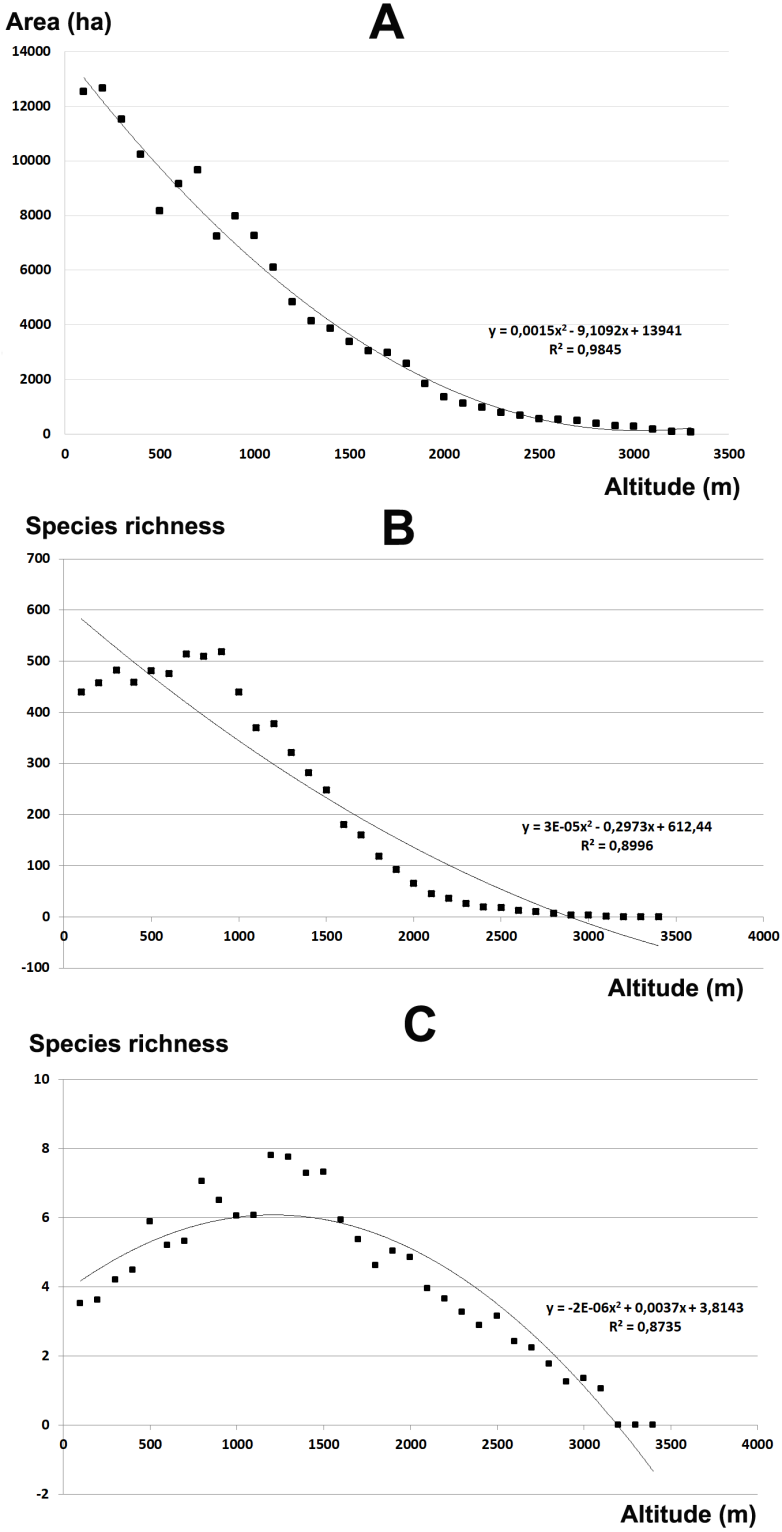
Elevational gradient of area and vascular plant species richness in Etna. (A) Area for each elevational belt; (B) number of species for each altitudinal interval; (C) distribution of the floristic richness in relation to the real surface of each belt, expressed as a percentage.

Overall, with increasing altitude, the area for each altitudinal belt declines almost constantly (Fig. 3A). The plant species richness shows an increase up to 1,000 m a.s.l. followed by a strong decline (Fig. 3B), while in relation to the belt area expressed in percentage (Fig. 3C) shows a *hump-shaped* response with a peak at 1,200–1,500 m a.s.l. From 1,500 to 3,300 m an unceasing reduction of the plant species richness occurs. Moreover, a strong correlation between area of each altitudinal belts and total species richness is observed (*r* = 0.96).

**Figure 4:**
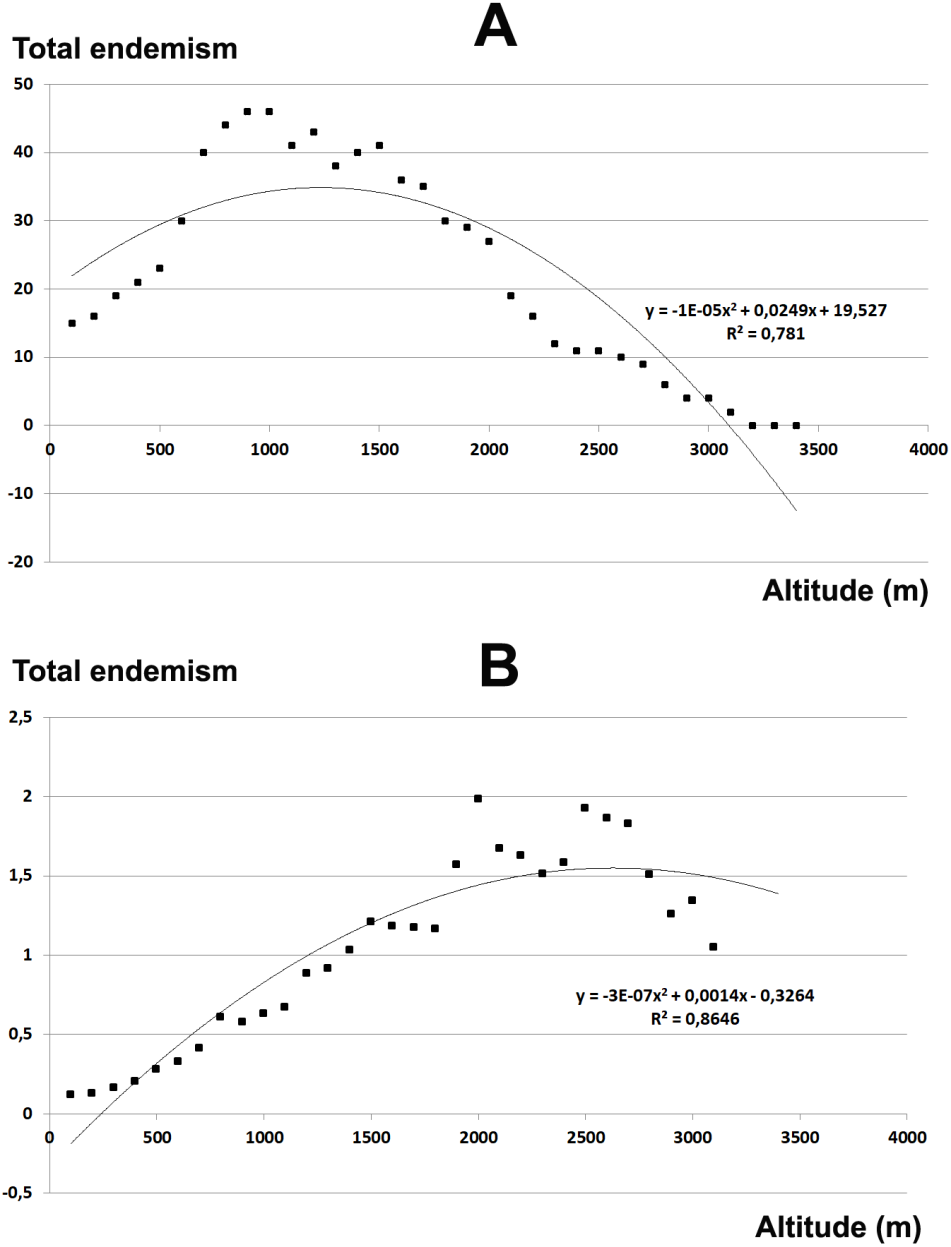
Elevational gradient of endemic species in Etna. (A) Number of endemic species for each altitudinal interval; (B) Distribution of endemic species in relation to the real surface of each belt, expressed as a percentage.

**Figure 5:**
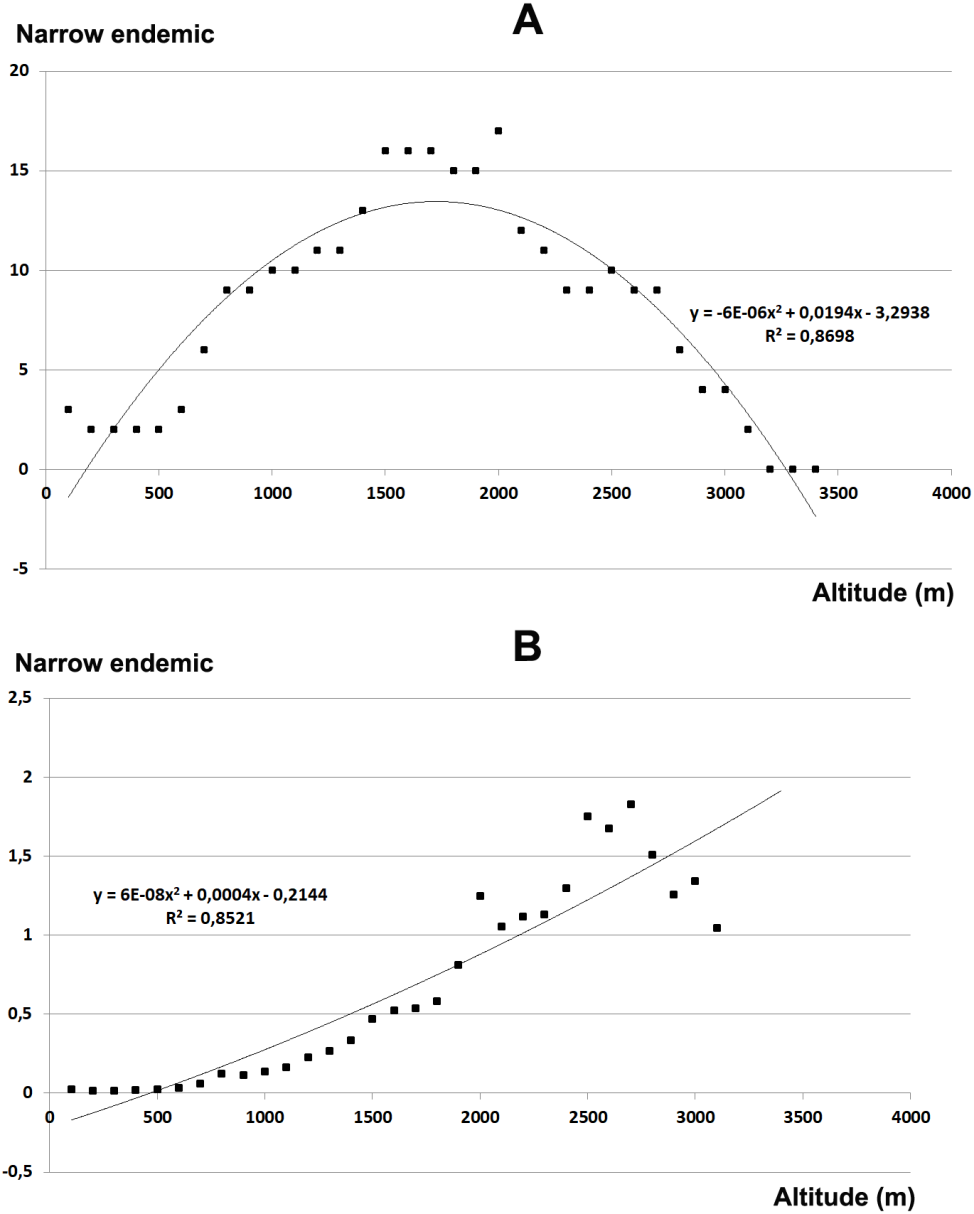
Elevational gradient of narrow endemic species in Etna. (A) Number of narrow endemic species for each altitudinal interval; (B) distribution of narrow endemic species in relation to the real surface of each belt, expressed as a percentage.

The total endemic species richness shows a significantly *hump-shaped* response to elevation gradient with a peak between 700–1,500 m a.s.l. (Fig. 4A), while in relation to the area of each altitudinal belt (Fig. 4B), shows an *increasing response* positively correlated with the elevation, with the highest values between 2,000–2,800 m a.s.l. The narrow endemic species (EE) have an *hump-shaped* response to elevation with a peak between 1,500–2,000 m a.s.l. (Fig. 5A), while in percentage the diagram shows a sharp *increasing response* with the elevation gradient, with a peak between 2,500–2,800 m a.s.l. (Fig. 5B). We observe a strong correlation between the area of each altitudinal belt and the endemic plant richness (*r* = 0.94), while no correlation with narrow endemic (*r* = 0.29). Moreover, there is a high correlation between species richness and total endemic species (*r* = 0.78).

**Figure 6:**
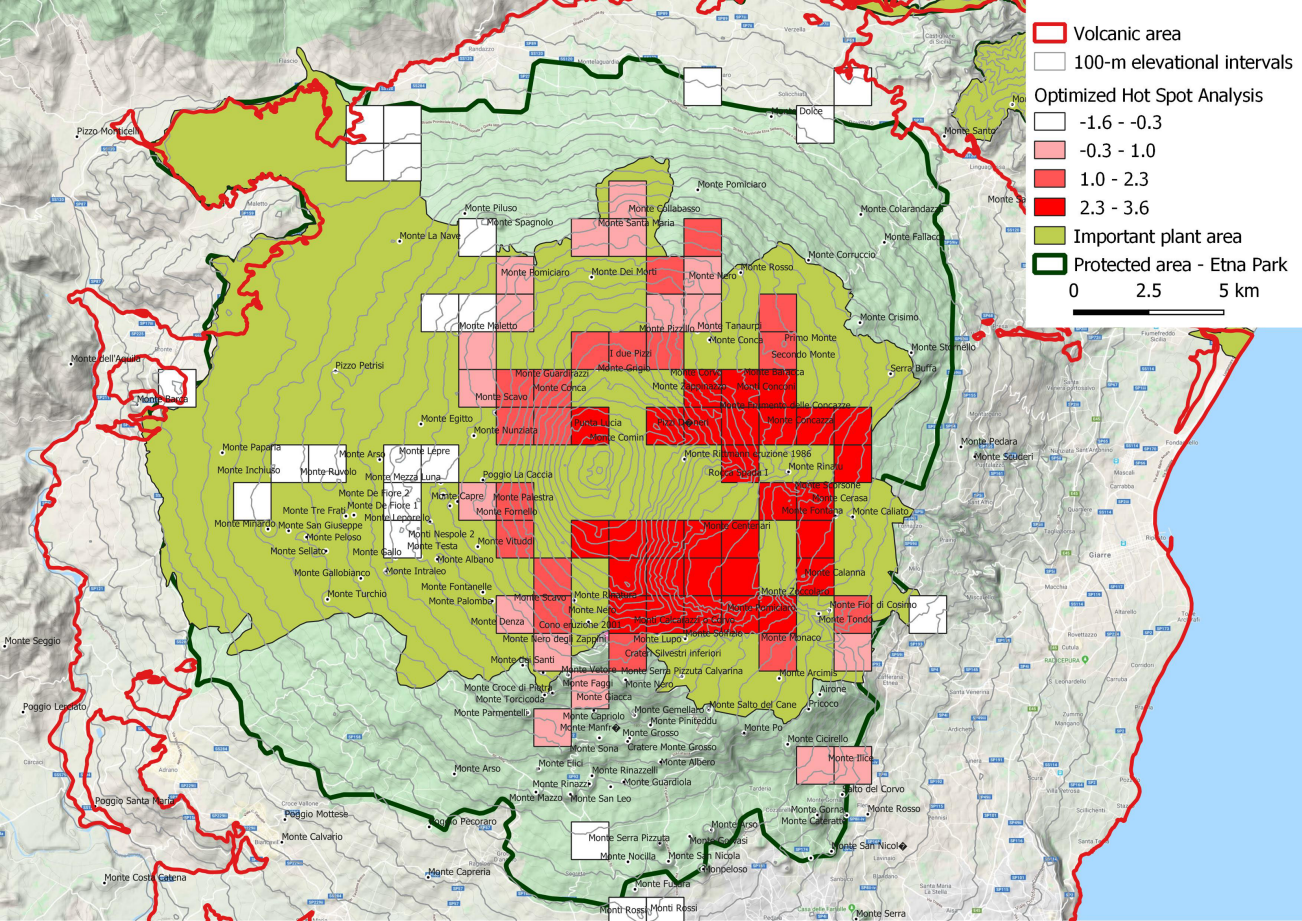
Spatial *Z*-score map, indicating the clustering features (Getis-Ord Gi*) of narrow endemic species (EE). Positive Gi* Z-scores denoted the spatial dependence of high values, while negative Gi* *Z*-scores indicated the spatial dependence of low values. Hotspot squares with a side of 1,250 m. This is an important plant area according to Blasi et al. (2010).

### Hotspots analysis of narrow endemism species

The spatial *Z*-score map (Fig. 6) was obtained processing the narrow endemic species distribution (EE). The areas with high concentration of narrow endemic species, are in correspondence of Oro-Mediterranean (1,800–2,400 m a.s.l.) and Cryo-Mediterranean (2,400–2,800 m) bioclimatic belts. In these bioclimatic belts, the species have an uneven distribution, with a marked preference for the east portion of the volcano. This area corresponds to one of the most active areas of the volcano especially for the fallout of volcanic ash and only partly for volcanic eruptions. It is important to note this area also includes the older high altitude volcanic substrates bordering the depression of the Bove valley. In a remarkable variety of natural rocky habitats, here grow some of the most singular species find refuge, as *Adenocarpus bivonii*, *Anthemis aetnensis*, *Astragalus siculus*, *Betula etnensis*, *Hieracium pallidum* subsp. *aetnense*, *Senecio aethnensis*, etc.

## Discussion

### Degree of species richness and endemism

Vascular flora richness of Mt. Etna roughly corresponding to one third of the Sicilian flora, while the endemic component corresponds to more than a fifth of Sicilian endemism. In fact, in Sicily grow about 415 endemic taxa corresponding to 12.7%, of the overall flora of 3,252 taxa (*Giardina, Raimondo & Spadaro, 2007; Raimondo, Domina & Spadaro, 2010*). Despite its recent genesis and unceasing volcanic activity, the endemism degree recorded for Mt. Etna is remarkable but smaller than that of other Mediterranean mountain areas of comparable size (Table 1). Based on the Spearman coefficient applied to the data in Table 1, we observe a high correlation between species richness of these Mediterranean territories and their endemic species (*r* = 0.78). Moreover, there is a strong correlation between species richness/endemic species and area (*r* = 0.88, *r* = 0.75), while *r* = 0.65 between narrow endemic species and area. Overall, the Etnean endemism highlights once again the floristic uniqueness of the Mediterranean high mountains, explained by geographical, ecological isolation, and geological history (*Trigas, Panitsa & Tsiftasis, 2013; Jeanmonod et al., 2015; Buira, Aedo & Medina, 2017*).

Actually, Mt. Etna can be defined as a peculiar “threefold island”, due its geo-pedological uniqueness in the Sicilian context, altitudinal development, and geographical isolation. Formerly, also *Kallimanis et al. (2010)*, showed for the Aegean archipelago, where geographic isolation plays an important role, the endemism strongly correlated with the floristic richness. *Emerson & Kolm (2005)* achieved the same result using data of plants and arthropods of the volcanic archipelagos of the Canary and Hawaiian Islands. In agreement with *Mahdavi, Akhani & Van der Maarel (2013)* the extremely narrow distribution range of the Etna’s endemics is due the to historical, geological, environmental features, as well as to species-specific parameters, e.g., habitat specialization, reproductive biology, dispersal ability, and so on.

### Life form patterns

Life form is considered a fundamental plant species strategy in response to the hydro-thermic gradient (*Wang et al., 2002; Mahdavi, Akhani & Van der Maarel, 2013*). On the whole, the endemic flora of Mt. Etna is characterized by the preponderance of the hemicryptophytes and geophytes, while rather low are the occurrence of the therophytes and chamaephytes, although these latter become important in the high mountain range. This biological form is the one that best adapts to cold climate conditions and is also able to counteract the volcanic disturbance mainly represented by the frequent falls of volcanic ash and lapilli which often completely cover the plant. Thanks to their powerful root system, it can re-emerge from the ash cover and regenerate the vegetative surface. The speciation and survival of endemic species, as already highlighted by other authors (*Médail & Verlaque, 1997; Soriano et al., 2012*) is probably favored by the presence of selective habitats such as those of high altitude. The dominance of hemicryptophytes in high mountain was also confirmed in the endemic flora of Corsica (Verlaque, Médail & Aboucaya, 2001; Jeanmonod et al., 2015), Greece (Georghiou & Delipetrou, 2010) and Sardinia (Bacchetta, Iiriti & Pontecorvo, 2005).

**Table 1:** Plant richness and endemic *taxa* of some Mediterranean lands.

Region	**Plant species**	**N. of endemics**	**Endemism (%)**	**N. of restricted endemics**	**Restrictedendemics (%)**	**Surface (Km^2^)**	**Highest peak altitude (m)**	**Authors**
Balearic Islands	1,500	180	12	144	9.6	4,992	1,432	Médail & Verlaque (1997)
Corsica	2,680	284	10.6	122	4,5	8,680	2,706	Jeanmonod et al. (2015)
Sulcis (SW Sardinia)	1,235	93	7.5	18	1.4	2,130	1,113	Bacchetta (2006)
Aspromonte	1,295^a^	121	9.7	28	2.2	1,650	1,956	Brullo, Giusso del Galdo & Guarino (2001)
Etna	1,055	92	8.7	29	2.7	1,370	3,328	this study
Hyblaean district	1,527^a^	105	6.9	20	1.3	4,730	986	Brullo et al. (2011)
Peloritani district	1,220^a^	128	10.5	15	1.5	1,827	1,350	Sciandrello et al. (2015)
Cyprus	1,620	171	10.6	158	9.8	9,251	1,952	Alziar (1995)
Crete	1,852	352	19	194	10.5	8,265	2,456	Trigas, Panitsa & Tsiftasis (2013)

**Notes.**

a subspecies included.

The predominance of annual plants at low altitudes, in agreement with Mahdavi, Akhani & Van der Maarel (2013), partly can be explained by soil and vegetation disturbance by human pressure. Another factor that explains the spread of the annual species at low altitudes are the quite high average of the annual temperatures, around 16−18 °C. The annuals are disadvantaged by very low average of annual temperatures and therefore cannot colonize the high mountain areas of the volcano, characterized by long periods of snow cover and strong freezing winds. Chamaephytes are instead the dominant life form in the upper zone (*Astragalus*-dominated plant community). The survival of chamaephytes is related to the rocky substrates with thin soil and strong climatic conditions (wind, snow, frost, etc.). The most peculiar, widespread and abundant especially in terms of biomass, among these species is *Astragalus siculus*, a narrow endemic chamaephytic species originated as a result of the spread in the Mediterranean area of the Irano-Turanian floristic element (*Pignatti et al., 1980*). This endemic low shrub is predominant between 2,000 and 2,700 m a.s.l., in agreement with Mahdavi, Akhani & Van der Maarel (2013) to Alborz Mountains (Iran). Hemicryptophytes and chamaephytes are life forms that go more in high altitude, localized, respectively, up to 3,000/3,100 m of altitude. These last two types represent the more competitive life forms at high altitudes because they can survive to unsuitable climatic and edaphic conditions like snow cover, strong winds and soil erosion or regeneration as occurs on Etna at high altitudies due to the frequent tephra fall.

### Elevational gradient of species richness/endemism

Area available for plants on high mountains is not constant through time for example due to the climatic changes of the past, but also of the future, which can cause altitudinal and latitudinal shifts of plant species and local extinctions (Normand et al., 2011). In the case of an active volcano the area variations could be much more relevant and frequent because lava flows or tephra falls may suddenly cover huge surfaces (e.g., up to 40 km^2^ during the 1,669 eruption –*Branca et al., 2015*) or catastrophic natural events may reduce available area as a consequence of huge collapses as happened 15.000 years ago on Etna. In addition, such variations may mainly affect areas at high elevation and, subsequently, cause variations in total plant species richness, degree of isolation and thereby the extent of endemism.

On the basis of these premises, the endemism of a volcanic mountain should be low and indeed the Etna degree of endemism, compared with other nearby areas is lower, however it remains important in number and with a prevalence of exclusive endemism at high altitudes. The occurring of endemic species on Etna mountain top is probably linked to its isolation in a context of nearby low mountains (lower than 2,000 m above sea level). This type of isolation is considered an important driver of speciation because gene flow is reduced (*Steinbauer et al., 2016*). There is also support from phylogenetic studies for an increase in diversification with elevation (*Merckx et al., 2015*), particularly in high elevation ‘island-like habitats’ (*Hughes & Eastwood, 2006*). Moreover, phylogenetic evidence indicates that many high-elevation endemics across the globe are phylogenetically young taxa resulting from recent fast diversification (*Winkworth et al., 2004; Merckx et al., 2015; Walter et al., 2020*). This fact fits well with the geological evolution of Etna, which, although started almost 600,000 years ago on the sea floor, really a true stratovolcano (named “Ellittico”) exceeding 3,000 m in altitude, is formed only from about 100,000 years ago (*De Beni et al., 2011*). This volcanic edifice due to several plinian eruptions (*Coltelli, DelCarlo & Vezzoli, 2000*) collapses about 15,000 years ago and from that period starts building the current volcanic edifice. The times of differentiation and speciation for altitude plants has been therefore rather short. In the same period, the glaciations overlap to these important volcanic activities (*Stewart, 2018*). The consequent climatic changes in the Mediterranean area and in particular the last Glacial period occurred from the last interglacial encompassing the period 115,000–11,000 years ago, but with alternation of cold period followed by temperate phases; the coldest period in absolute, started around 30,000 years ago (Ivy-Ochs et al., 2008). These changing environmental conditions, and the associated shifts of species, may have repeatedly divided and merged populations at different elevations, enhancing conditions favorable to speciation (*Qian & Ricklefs, 2000; Sandel et al., 2011; Gillespie & Roderick, 2014; Steinbauer et al., 2016*).

This mechanism fits well for the Mediterranean mountains, and therefore for Mount Etna, as the extinctions, which were responsible for the recent floristic impoverishment in Central Europe in the glacial periods, had less effect in the Mediterranean region enabling many taxa to seek refuge there (*Hewitt, 1999*). By providing suitable habitats during cold climatic periods, the refuge areas would have limited species extinction as well as favouring the emergence of new taxa (*Hungerer & Kadereit, 1998; Médail & Diadema, 2009; Buira, Aedo & Medina, 2017; Puglisi et al., 2019*).

This is the case of some tree species, such as *Fagus sylvatica* having a very high haplotype diversity revealing past fragmentation events that may have been occurred during the Quaternary glaciations with almost all haplotypes remained trapped in the Italian peninsula and Sicily, with southernmost population on Etna (*Vettori et al., 2004*). In other cases, more pronounced isolation and fragmentation led to the onset of endemic species such as *Betula etnensis* an isolated little population on Etna Mount, about 500 km away from the southernmost Italian population of *Betula pendula* from which it probably separated (*Bagnato et al., 2014; Tsuda et al., 2017*). On the other hand, Etna, despite its young age, is also a refuge for very ancient species that, disappeared in the rest of Sicily, have found refuge in its high mountain area. The most emblematic case is that of *Pinus nigra* subsp. *calabrica*, a typical element of the Calabrian Peloritan arch (*Cirrincione et al., 2015; Sciandrello et al., 2015*); it marks an ancient geological history also linked to the Sardinian-Corsican plaque (Bonavita et al., 2016). Today *P. nigra* subsp. *calabrica* is absent in Peloritani Mounts mainly because of their too low altitude. In the Nebrodi Mountains the presence, even if modest, of *Pinus* pollens at the end of the ice age is documented (*Bisculm et al., 2012*), this occurrence is also better registered at Pergusa Lake in Erei Mountains (*Sadori & Narcisi, 2001*) where it was quite abundant about 10.000-9200 years ago. Also *Genista aetnensis,* endemic of Sicily and Sardinia, is probably a tertiary relict (*Arrigoni & Vannelli, 1967*), testifying ancient geographical connections, widespread in Sicily as native only on Etna where it found optimal habitat for its establishment. Overall, Etna, the only mountain having an important height in Sicily, had a backwash effect on many species of cold climate that elsewhere in Sicily could no longer survive and some of these had the opportunity to undergo to speciation processes contributing to the arising of Etna endemism.

### Hotspots analysis of narrow endemism species

The hot spot analysis here performed, gives indications on less obvious drivers of narrow endemism, assuming that the high altitude and the resulting isolation are the main ones, as is now universally accepted (Steinbauer et al., 2016). It is observed, first of all, making a comparison with the geological map of Branca et al. (2011), that the hottest areas are concentrated over, or near, the oldest emerging volcanic substrates of high altitude such as the internal and external flanks of “Valle del Bove”. This could mean that the oldest volcanic rocks (110–15 ka) functioned as refuge and differentiation areas of high altitude volcanic environment species. On the other hand, a good correspondence of hot spot areas with the prevailing deposition of volcanic tephra and ash is also observed. *Scollo et al. (2013)* stated that the prevailing wind direction in the last millennia favored the fall of tephra and ashes on the east flank of Etna, with maximum concentration precisely in the hot areas of endemism. In some ways this is an opposed driver to the previous one: a continuous regeneration of the superficial soil stimulates the plant recolonization and the possible evolutionary differentiation of the colonizers. But these opposite tendencies express the need for plant organisms in having refuge areas where they can survive and, at the same time, new young soils on which experimenting colonizing capacities and rapid adaptation to changing environmental conditions.

Moreover, the hot spot analysis could give indications on the protection of endemisms to the managing body of the natural park but however this is already the most protected area of the volcanic complex as it falls entirely in A zone (area with the highest protection level) of the park.

## Conclusions

The vascular plant richness of the Etna Mountains discussed in this paper highlighted the floristic value of this volcano and its unique role in the context of Sicily. This contribution wanted to go one further step by using quantitative data on the spatial distribution of the plant species along the altitudinal gradient and highlight areas with a high concentration of narrow endemic species. In this way, it has been possible to highlight the remarkable altitudinal variations in the species distribution, which is less obvious than one might think; in particular, the peculiar ”hump-shaped pettern” of floristic richness at medium altitudes was highlighted corresponding where both thermophilic and cooler species can find favorable environmental conditions. In relation to the area of each altitudinal belt, the total endemic and narrow species show an increasing response correlated with the elevation. The high degree of endemic species on the highest peaks of Mt. Etna is linked to its geographical, geological and climatic isolation, all important drivers of speciation acting on the population gene flows. The endemism despite having such peculiar and exclusive species to Etna, on the whole shows lower numerical values than other nearby floristic districts that are not volcanic but comparable in size and altitude. A plausible explanation is that the geological substrate youth and the frequent destruction of the plant communities following the lava flows have contributed to selecting a restricted number of species well adapted to these peculiar conditions. Etna as a whole remains unique in the Mediterranean and for this reason it has not been possible to make comparisons with the flora of the active volcanoes of this region, all considerably smaller and of much lower altitude.

##  Supplemental Information

10.7717/peerj.9875/supp-1 Supplemental Information 1 Vascular flora of Mt. Etna Floristic list with indication of the Family, Taxon, Life form, Endemism type and altitude range.

## Additional Information and Declarations

### Data Availability

The following information was supplied regarding data availability: Raw data is available as a Supplementary Files.
